# Evaluation of Anaesthetic Approaches in Transcatheter Aortic Valv Implantation Procedures

**DOI:** 10.4274/TJAR.231270

**Published:** 2023-10-24

**Authors:** Murat İzgi, Adem Halis, Yusuf Ziya Şener, Levent Şahiner, Ergün Barış Kaya, Kudret Aytemir, Ayşe Heves Karagöz

**Affiliations:** 1 Department of Anaesthesiology and Reanimation, Faculty of Medicine Hacettepe University, Ankara, Turkey; 2 Department of Cardiology, Faculty of Medicine Hacettepe University, Ankara, Turkey

**Keywords:** Aortic stenosis, general anaesthesia, perioperative care, sedation, transcatheter aortic valve implantation (TAVI)

## Abstract

**Objective:**

Transcatheter aortic valve implantation (TAVI) has emerged as an alternative to surgical aortic valve replacement and has become a popular treatment modality for inoperable or patients at high surgical risk with severe aortic stenosis. We aimed to evaluate our perioperative anaesthetic experiences with patients undergoing TAVI under sedation or general anaesthesia (GA).

**Methods:**

One hundred and fifty-nine patients who underwent TAVI procedures were enrolled. Effects on TAVI outcomes of sedation and GA were compared.

**Results:**

The duration of surgery and anaesthesia was significantly longer in patients who received GA. Insertion site complication and post-TAVI pacemaker implantation rates were similar between the groups, but the frequency of intraoperative complications (10% vs. 0.8%; *P*=0.015), intraoperative hypotension (35.3% vs. 70%; *P* < 0.001), and acute kidney injury (12.6% vs. 27.5%; *P*=0.028) was significantly higher in the GA group. Stroke occurred in seven patients, and all were in the sedation group.

**Conclusion:**

GA is related to increased procedure time and acute kidney injury; therefore, local anaesthesia and sedation may be the first option in patients undergoing TAVI.

**Main Points**

• Evaluation of perioperative anaesthetic experience with patients undergoing transcatheter aortic valv implantation (TAVI) under sedation or general anaesthesia (GA) is aimed.

• Using local anaesthetics with sedation is a more popular modality than GA with endotracheal intubation due to the advantages of sedation.

• Acute kidney injury is a frequent complication after TAVI.

• Local anaesthesia with sedation can be safely performed during transfemoral TAVI procedures.

## Introduction

Aortic stenosis is a common valvular disease and valvular replacement is required in symptomatic patients with severe aortic stenosis. Surgical aortic valve replacement is the gold standard treatment in patients who are fit for surgery, but a significant proportion of patients carry high perioperative mortality risk or refuse surgery. Transcatheter aortic valve implantation (TAVI) has emerged as an alternative option to surgical aortic valve replacement in recent years and has become a popular treatment modality for inoperable or patients at high surgical risk with severe aortic stenosis. The procedure should be performed with general anaesthesia (GA)/sedation under the guidance of anaesthesiologists for patient safety and comfort. The choice of anaesthesia technique for TAVI may vary depending on the nature of the selected procedure, the additional comorbidities of the patient, and the experience of the team performing the procedure. GA is advantageous for respiratory control, patient immobility, hemodynamic stability, enabling the use of transesophageal echocardiography (TEE), facilitating the management of procedural complications, and arterial interventions. As the experience of the team increases, the choice of a local anaesthesia-sedation (LAS) combination becomes more prominent. The LAS combination has advantages such as the early detection of neurologic complications, short procedure time, rapid recovery, and reduced postoperative care requirements. The main limitations of sedation are the invasiveness of the procedure and the difficulty of obtaining stable hemodynamics.^1,2^

We aimed to evaluate our perioperative anaesthetic experience with patients undergoing TAVI under sedation or GA.

## Methods

After obtaining approval from the Hacettepe University Non-Invasive Clinical Research Ethics Committee (approval no: 2019/24-16, date: 15.10.2019), a retrospective review of anaesthesia management data for TAVI procedures was conducted. All patients (n = 159) who underwent percutaneous TAVI procedures in our hospital from 2013 to 2018 were included in the study.

Patients’ characteristics such as age, sex, comorbidities, and data including echocardiographic parameters, anaesthesia method, anaesthetic drugs used, surgical duration, hospitalization time in the cardiac intensive care unit (ICU), and total hospitalization time, perioperative complications, and mortality were recorded for all patients. Patients’ perioperative mortality risk was assessed using the Society of Thoracic Surgeons and Logistic EURO score models.

The pre-anaesthetic evaluation was performed one to two days before the procedure. Following a fasting period of 8 hours, standard monitoring with a 5-lead electrocardiogram, non-invasive blood pressure, pulse oximeter, and capnography were performed on all patients. After the femoral arterial sheath side port was placed, invasive arterial blood pressure was also monitored. GA was induced with intravenous (IV) anaesthetics from the IV catheter placed and the maintenance of GA was provided with 2% sevoflurane in a 50% oxygen - N_2_O mixture through an anaesthesia device (Datex Ohmeda ADU S - 5, Finland). Induction for sedation was performed with midazolam and fentanyl and the maintenance of sedation was provided with iv infusion of propofol with bolus doses of ketamine or fentanyl if needed.

The time from induction of anaesthesia to tracheal extubation was recorded as the anaesthesia time for patients who underwent the procedure under GA. The duration of anaesthesia for patients who underwent the procedure under LAS was defined as the time from the administration of sedative agents until the decision to take the patient out of the operating room. The time from the initiation of vascular cannulation by the cardiologist to the removal of the catheters was recorded as the surgical time.

We aimed to evaluate and compare outcomes in patients who underwent TAVI under either GA or sedation. Outcomes were defined as periprocedural complication rates and mortality rates. Periprocedural complications were defined as insertion site complications, stroke, permanent pacemaker implantation, acute kidney injury (AKI), and intraoperative hypotension. The AKI was identified as an increase in creatinine level of 1.5-1.9 times that of baseline or ≥0.3 mg dL^-1^ ( ≥26.5 mmol L). Intraoperative hypotension was defined as a mean arterial pressure <65 mmHg for at least one minute.

All statistical analyses were performed using the Statistical Package for the Social Sciences (SPSS) version 22.0 (SPSS, Inc., Chicago, IL) statistical software. Mean, standard deviation, median, lowest, highest, frequency, and ratio values were used in the descriptive statistics of the data. The distribution of variables was measured using the Kolmogorov-Smirnov test. The Mann-Whitney U test was used for the analysis of quantitative independent data. A chi-square test was used for the analysis of qualitative independent data.

## Results

A total of 197 patients were screened and 159 patients (58 males, 101 females) with accessible data were included. The mean age of the study population was 76.6±10.1 years. Hypertension was present in 119 (74.8%) patients and diabetes was present in 46 (28.9%). Baseline characteristics are presented in Table 1. The median follow-up after TAVI was 36.3 (0-77.4) months. All procedures were performed under deep sedation or GA. GA was preferred in 40 (25.1%) patients. Twenty-seven (17%) patients were intubated via an endotracheal tube and a laryngeal mask airway was used in 13 (8.2%) patients. The most used IV agents during the procedures were midazolam (94.3%) and propofol (83%). The median surgical time was 70 (30-270) minutes and the median anaesthesia time was 80 (45-300) minutes. The procedure was completed without any complications in 154 (96.8%) patients. Five (3.1%) patients had intraoperative complications including cardiac arrest (n = 3), femoral artery injury requiring surgery (n = 1), and ventricular rupture (n = 1). One of the patients with cardiac arrest died. Procedural characteristics are presented in Table 2.

Patients were classified into two groups according to the performed anaesthesia modality; GA or deep sedation. Baseline characteristics were similar between the groups except for hemoglobin (Hb) levels (Table 1), which were significantly lower in the GA group (12.1±1.8 vs. 11.2±1.7 g dL^-1^ *P*=0.008). Midazolam, fentanyl, and ketamine use were significantly higher in the sedation group, whereas remifentanil use was higher in the GA group (Table 2). Anaesthesia duration, surgical duration, amount of given fluid, and intraoperative complication rates were higher in patients who underwent GA compared with sedation (Table 2).

**Figure 1:**
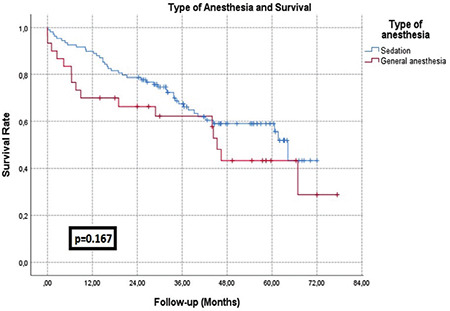
The survival curves of the sedation group and general anaesthesia group.

The median length of ICU stay was higher in the GA group (6 vs. 9.5 days; *P*=0.047), but the length of hospital stay was similar between the groups. Insertion site complications and post-TAVI pacemaker implantation rates were similar between the groups, whereas the frequency of intraoperative complications (0.8% vs. 10%; *P*=0.015), intraoperative hypotension (35.3% vs 70%; *P* < 0.001), and AKI (12.6% vs. 27.5%; *P*=0.028) was significantly higher in the GA group. Stroke occurred in seven patients, and all were in the sedation group. In-hospital (1.7% vs. 10%; *P*=0.035) and all-cause mortality rates (35.3% vs. 52.5%; *P*=0.045) were higher in the GA group (Table 3). However, we realized that a significant proportion of deaths occurred in the earlier patients as GA was preferred more in the preliminary cases. Therefore, we thought that it might be due to the effect of learning curve and we reassessed the survival analysis after excluding the patients who took place in the first 30 cases of our cohort.  Ten cases in the GA group and 11 cases in the sedation group were excluded. In addition, 9 of these 30 patients were in the excluded group because their data could not be reached. Both in-hospital and overall all-cause mortality rates were similar between the two arms (Table 3). Survival was assessed using Kaplan-Meier analysis curve and survival chart (after the learning curve affect eliminated) is presented in Figure 1.

## Discussion

Recently, the TAVI procedure is more preferred over surgical aortic valve replacement for high-risk patients with symptomatic and severe aortic stenosis.^3,4^ The anaesthetic management of patients undergoing TAVI has become more important nowadays. Using local anaesthetics with sedation is a more popular modality than GA with endotracheal intubation due to the advantages of sedation.^1,2,5,6,7^ In our study, sedation was more preferred than GA during TAVI procedures in accordance with the literature. The use of GA was reduced over time due to the increasing experience of the team in our unit, as described in the literature. If TEE should not be performed or there are no other indications for GA with endotracheal intubation during TAVI procedures, sedation is a good alternative for this process. It was shown that procedures performed with sedation were related to shorter-duration surgical time, anaesthesia time, hospital and ICU stays, and a lower incidence of respiratory complications and hypotension than with GA.^6,7,8^ The results of our study are compatible with the literature.

The safest environment for TAVI procedures is a hybrid operating room that includes imaging equipment for faster intervention if surgical intervention is needed during the procedure. In many centers, TAVI procedures are performed in a cardiac catheterization laboratory (CCL). In our center, TAVI procedures are currently performed in a CCL, but it is close to the cardiac surgery operating room. The anaesthetist should have all the critical drugs and equipment required for intervention should any emergency condition occur due to complications of the procedure. In our center, we have all the drugs we would need in an emergency in a Pyxis unit and all anaesthetic equipment in the CCL.

In the selection of anaesthetic agents to be used for GA and sedation, it is recommended to use drugs that ensure the stability of hemodynamics, generally comprising agents such as etomidate, propofol, and ketamine.^9^ Moderate-acting agents such as rocuronium can be used as muscle relaxants during intubation. It is emphasized that dose titration is more important than drug choice in keeping hemodynamics stable.^1^ In this study, we used midazolam for most patients to reduce the dose of other anaesthetic drugs to keep the hemodynamics stable. We used propofol infusion for sedation to achieve a stable drug plasma concentration.

Several studies compared the effects of anaesthesia methods on TAVI outcomes. It is reported in all of the studies that surgical time and hospital stay are longer in patients who undergo GA when compared with sedation, as in our study. There are controversial results regarding the effects of the anaesthesia method on in-hospital mortality rates after TAVI; however, it is expressed in all studies that the anaesthesia method does not affect mid to long-term all-cause mortality rates.^10^ Harjai et al.^11^ reported that in-hospital and all-cause mortality rates were similar among the patients who underwent TAVI under GA and sedation during a median 365 day follow-up. A review including 13 non-randomized trials and in-hospital mortality rates concluded to be similar between the two anaesthesia methods.^12^ In our study, we first found that in-hospital and all-cause mortality rates were higher in the GA group. However, we realized that a significant proportion of deaths occurred in the early period of our TAVI experience. The possible explanation for the diverse mortality rates between the anaesthesia type is the impact of the operator’s learning curve on outcomes. We used to prefer GA in the beginning stages of our TAVI experience and by the time we switched our approach to sedation. Therefore, the GA group mostly underwent TAVI in the early experience period, which might have resulted in higher mortality rates. It is reported that operators performing TAVI need about 30 cases to become “better” and the cut-off value was determined as 30 cases.^13^ Hence, we excluded the cases who were included in the first 30 cases of our TAVI cohort, and we reassessed the mortality analysis after eliminating the learning curve effect. There were not any differences in both overall all-cause and in-hospital mortality rates between the two-anaesthesia type. After interpreting these results, our findings suggest that anaesthesia type has no impact on mortality rates after TAVI as compatible with the previous data.

Intraoperative hypotension is defined as having a mean arterial pressure <65 mmHg for at least one minute. It is shown that intraoperative hypotension is associated with an increased risk of postoperative AKI and mortality.^14,15^ In our study, intraoperative hypotension occurred in 70 (44%) patients, the frequency of intraoperative hypotension was significantly higher in the GA group despite left ventricular dimensions and wall thickness parameters being similar between the groups.

AKI is a frequent complication after TAVI and it is reported in ranges from 8.3% to 58%.^16,17^ There are several defined risk factors for post-TAVI AKI including contrast media volume, red blood cell transfusion, post-procedural leukocyte count, peripheral artery disease, and intraoperative hypotension.^18^ In our study, AKI developed in 26 (16.4%) patients and significantly more in the GA group. This finding might be due to higher rates of intraoperative hypotension and the lower Hb levels in the GA group.

Perioperative stroke is defined as a cerebral infarction that develops during or after an interventional procedure, with the postoperative period being up to 30 days.^19^ In our study, stroke occurred in 7 (4.4%) patients, and all were in the sedation group. This finding should not be interpreted as a clinically significant result because all the strokes occurred more than 1 month after the procedures and despite it not being statistically significant, follow-up was longer in the sedation group. We also did not evaluate the patients’ preoperative stroke-related risk factors such as the presence of atrial fibrillation.

Due to the retrospective design of the study, we could not obtain some data including the contrast media volume used, American Society of Anesthesiologists (ASA) scores, and causes of mortality. Using propensity score matching could have extinguished the effects of confounding factors on outcomes and would be better preferred; however, the absence of significant differences in age, gender, and comorbidities among both groups reduces the impact of these confounding factors on outcomes.

## Conclusion

TAVI is a great option for patients with severe aortic stenosis who are at high risk for surgical repair. We observed that LAS can be safely performed during transfemoral TAVI procedures and may be an appropriate option in these patients. The anaesthesia method should be selected according to the medical condition of the patient and the experience of the team; however, LAS may be the first option in suitable patients due to the shorter surgical duration. We believe that close follow-up, dose titration, and equipment preparation are important for both methods, and caution is needed in terms of complications that may develop intraoperatively.

## Figures and Tables

**Table 1:**
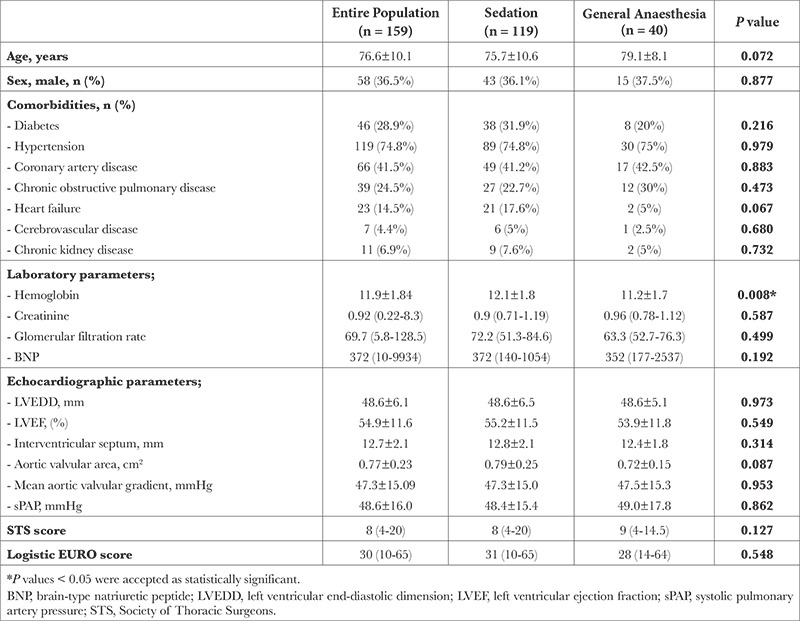
Baseline Characteristics

**Table 2:**
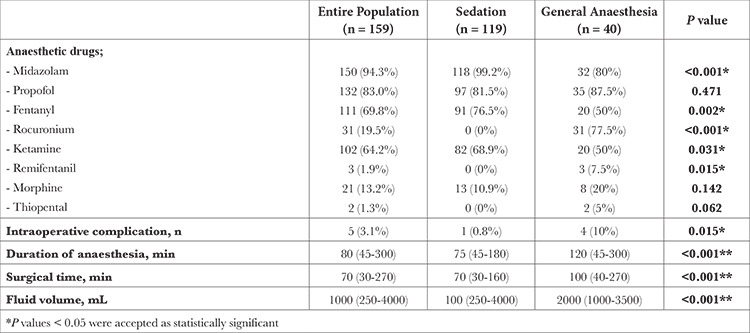
Procedural Characteristics

**Table 3:**
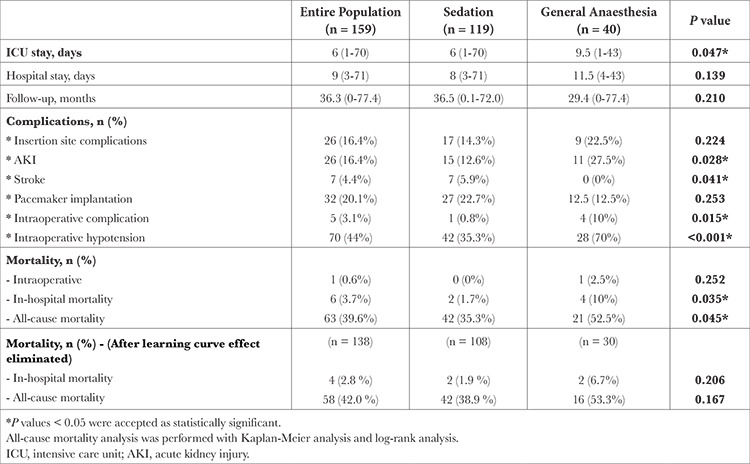
Follow-up and Outcomes
